# Orthostatic proteinuria due to inferior vena cava interruption without nutcracker phenomenon in an old obese female: a case report and literature review

**DOI:** 10.1186/s12882-023-03279-y

**Published:** 2023-07-31

**Authors:** Liling Lin, Kai Zhang, Xiao Yang, Lu Lin, Xuemei Li, Ling Qiu

**Affiliations:** 1 Department of Laboratory Medicine, State Key Laboratory of Complex Severe and Rare Diseases, Peking Union Medical College Hospital, Chinese Academy of Medical Sciences, No. 1 Shuaifu Yuan, Dongcheng District, Beijing, 100730 PR China; 2 Department of Ultrasound, Peking Union Medical College Hospital, Peking Union Medical College & Chinese Academy of Medical Sciences, Beijing, 100730 China; 3 Department of Radiology, Peking Union Medical College Hospital, Peking Union Medical College & Chinese Academy of Medical Sciences, Beijing, 100730 China; 4 Department of Nephrology, Peking Union Medical College Hospital, Peking Union Medical College & Chinese Academy of Medical Sciences, Beijing, 100730 China; 5 State Key Laboratory of Complex Severe and Rare Diseases, Peking Union Medical College Hospital, Peking Union Medical College & Chinese Academy of Medical Sciences, Beijing, 100730 China

**Keywords:** Orthostatic proteinuria, Inferior vena cava interruption, Left renal vein obstruction

## Abstract

**Background:**

Nutcracker syndrome (NCS) caused by left renal vein (LRV) entrapment, is one of the most common causes of orthostatic proteinuria. In stereotype, orthostatic proteinuria is often accompanied by left renal vein obstruction and is found in young and underweight individuals. Here, we report a rare case with orthostatic proteinuria in an old obese female caused by a rare type of congenital inferior vena cava (IVC) interruption.

**Case presentation:**

A 65-year-old obese woman, who suffered from fluctuated proteinuria, had been misdiagnosed as chronic glomerulitis for 30 years. Instead of having any sign of NCS, she had a unique type of IVC interruption. Most venous blood from infrarenal IVC and right kidney drained into her LRV, and then through the expanded communicating vessel, drained into the left ascending lumbar vein which extended as hemiazygos vein. To the best of our knowledge, this is one of the first cases reported of orthostatic proteinuria attributed to the subsequent hemodynamic irregularity caused by IVC interruption without nutcracker phenomenon.

**Conclusion:**

Adult-onset orthostatic proteinuria is relatively rare, hard to be recognized and could be misdiagnosed as chronic glomerulonephritis. The case provided a novel differential diagnostic condition for those who suffered from fluctuated proteinuria of unknown causes.

**Supplementary Information:**

The online version contains supplementary material available at 10.1186/s12882-023-03279-y.

## Background

Orthostatic proteinuria is characterised by elevated protein excretion in the ambulatory position and normal protein excretion in the recumbent position, first morning excretion, or 24-hour urine. Nutcracker phenomenon, often referring to left renal vein (LRV) entrapment, which is prevalent in underweight children and adolescent, is one of the most common causes of orthostatic proteinuria [1–3]. The mechanisms of orthostatic proteinuria were supposed to be related to exaggerated hemodynamic abnormality leading to altered glomerular permeability, on the basis subtle glomerular injury [4]. The LRV was most often compressed between the abdominal aorta and superior mesenteric artery or between the abdominal aorta and vertebral columns, or rarely by pancreatic neoplasms, retroperitoneal tumor, lordosis, and etc. [5]. Among the multiple types of inferior vena cava (IVC) abnormalities, left IVC and double IVC might cause nutcracker syndrome (NCS) [6]. In this article, we present a unique case of orthostatic proteinuria caused by increasing LRV pressure which was attributed to congenital IVC interruption without LRV entrapment. Here we will describe the clinical presentations, management, and renal outcome in details.

## Case presentation

A 65-year-old obese woman visited our hospital 2 years ago, with intermittent foamy urine and edema for over 30 years. The presence of foamy urine was apparent following daily activity, diminished in first morning excretion, and intensified during periods of excessive exertion. Her edema was mild to moderate, noticeable on the eyelids and double lower limbs, worsened in the morning, and alleviated or absent at night. Levels of urinary protein ranged from negative to over 3.0g/L, while red blood cells were insignificant in qualitative urinalysis. Albumin-to-creatinine ratio (ACR) fluctuated between 15 to 4131 mg/g (normal range: less than 30mg/g), while the 24-hour urinary total protein (24h UTP) was 0.05 to 0.25g/24h (normal range: less than 0.2 g/24h). The fluctuation of urinary tests from 2019 to 2020 were summarized in Additional file 1: Table S1. Sodium dodecyl sulfate-agarose gel electrophoresis for urinary protein analysis revealed the presence of 61.4% albumin, 13.2% transferrin, 20.9% IgG and 4.5% IgA, which indicated that the protein was originated from glomeruli. Her serum albumin was 39-48g/L (3.9-4.8 g/dL, normal range 35-52 g/L or 3.5-5.5 g/dL), serum creatinine was 54-77μmol/L (0.61-0.87 mg/dL, normal range 45-84μmol/L or 0.51-0.95 mg/dL), and other chemicals were all in normal range during these years. Urine sediment, C-reactive protein, erythrocyte sedimentation rate, autoimmune antibodies and immunofixation electrophoresis were all unremarkable.

The ultrasound examination revealed clear bilateral renal structure, with the left and right renal longitudinal diameters measuring 11.4 cm and 11.2 cm, respectively. Additionally, the blood flow in the superior mesenteric artery and bilateral renal veins was satisfactory. Past medical history included hypertension, with blood pressure well controlled via losartan potassium hydrochlorothiazide tablet. Other medical history included a substernal goiter and non-functional left adrenal adenoma, both of which had undergone surgical treatment successfully.

She did not receive renal biopsy because of normal 24h UTP and serum creatinine. Because of repetitive and constant spot urinary protein, the patient had been misdiagnosed as unexplained chronic glomerulitis by nephrologists and treated with traditional Chinese medicine, Shenyan Kangfu tablets, Huangkui capsule and Bailing capsule. The treatment led to no improvement, she was referred to multiple centers and consulted with different doctors, leading to redundant tests and unnecessary expenditure. After querying, we discovered that the patient collected 24-h urine sample at home with less activity, while the spot urine sample for routine urinalysis and ACR was often collected after she came to the hospital. The distance between her home and the hospital was 6 km, taking a total of 45 min of walking and bus ride. We asked the patient to collect urine each time she voided and to record the voiding time along with the corresponding activity status. As it’s depicted on Table 1, the patient's ACR was 3mg/g in first morning excretion, that elevated and fluctuated with daily activity. The routine was repeated four times which yielded similar results that the higher intensity of exercise, the more severe ACR and protein-to-creatinine ratio (PCR) in the urine. The data supported the diagnosis of orthostatic proteinuria. Additionally, there was a correlation between the severity of the patient’s edema and the ratio of ACR after commuting to the hospital compared to the ACR in the first morning excretion.

**Table 1:** Variation of ACR and PCR in a day

Time of Voiding		Activity	ACR, mg/g	PCR, mg/g
Day 1	8:00A.M	Sweeping floor	45	158
	9:20A.M	Doing housework	58	189
	11:07A.M	Rest	4	70
	12:30P.M	Standing 1 h	82	204
	14:37P.M	Rest	15	125
	18:30P.M	Rest	7	61
	20:00P.M	Jogging 1 h	413	654
	22:30P.M	Sleeping	15	59
Day 2	6:30 A.M	First excretion	3	43
	7:00 A.M	Second excretion	10	58
	8:00 A.M	Rest	23	93
	9:30 A.M	Coming to hospital	1356	1993

*ACR* albumin-creatinine ratio, less than 30 mg/g in normal condition, *PCR* protein-creatinine ratio, normally less than 200 mg/g

**Fig. 1:**
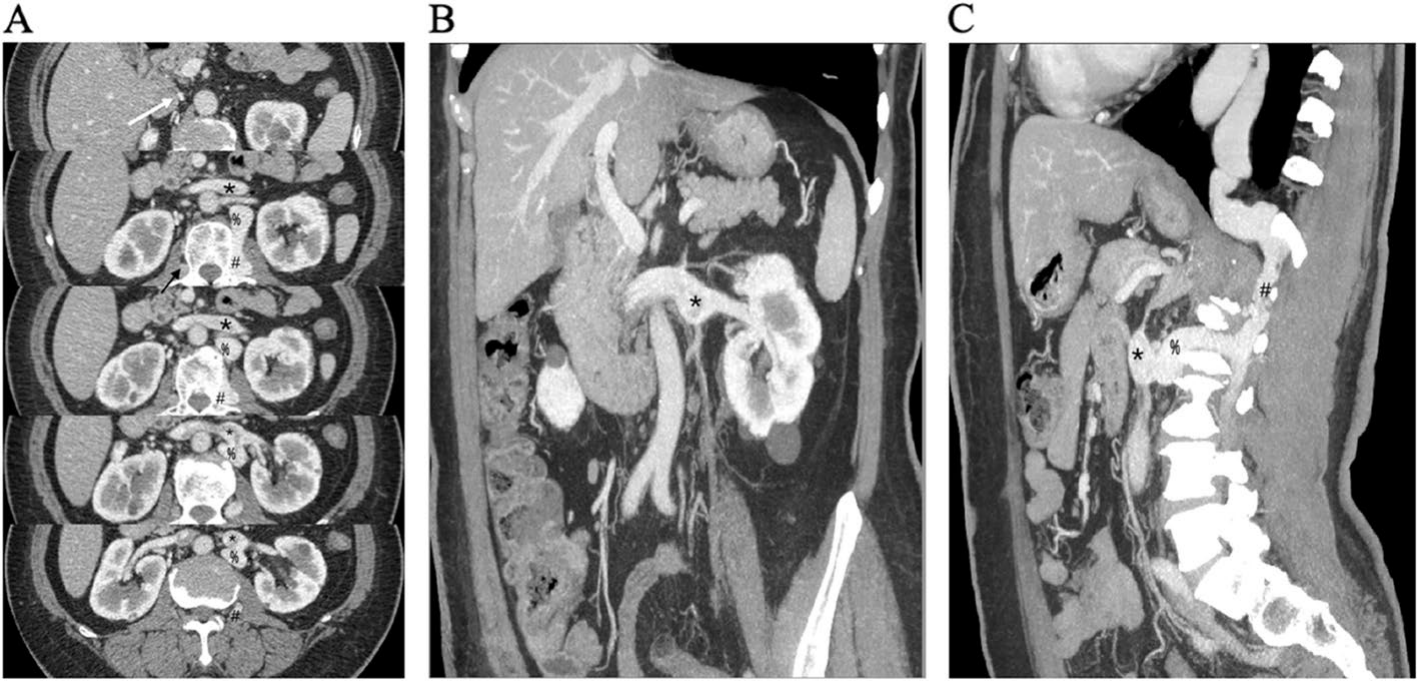
Contrast-enhanced abdominopelvic CT images of the patient in rest state. **A**. Cross-sectional images shows localized enlargement of LRV (*) and tortuous dilatation of the right ascending lumbar vein (black arrow), left ascending lumbar vein (#), and bilateral lumbar veins. There is no sign of nutcracker phenomenon. The white arrow indicates the blind end of infrarenal IVC. **B**. Oblique coronal MIP image at delayed phase shows local dilatation of left renal vein (LRV) (*) near the hilum. **C**. Sagittal MIP image at delayed phase shows that LRV (*) is connected with left ascending lumbar vein and hemiazygos vein (#) via a communicating branch (%), and all of the vessels are dilated. MIP, maximum intensity projection

LRV entrapment was suspected at first, but neither ultrasound nor abdominal computed tomography (CT) could find any signs of nutcracker phenomenon. Incidentally, the enhanced CT for her left adrenal tumor indicated she had the inferior vena cava (IVC) interruption between the subhepatic-suprarenal region, accompanied by a locally expanded LRV, as well as tortuous dilatation of the azygos vein, hemiazygos vein, and bilateral lumbar veins (Fig. 1).

**Fig. 2:**
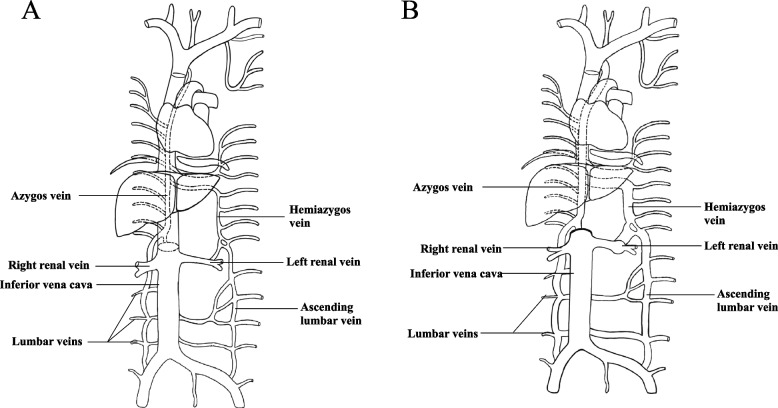
Schematic sketch of venous system. Picture **A** shows that the normal IVC is formed by the joining of the left and right common iliac veins and pours into right atrium. Picture **B** demonstrates the interruption of IVC from infra-hepatic to supra-renal segment. Different from previous cases whose IVC continue as azygos vein, our patient did not have the continuation. Her LRV received a large part venous blood from the infrarenal IVC and the right kidney, through an expanded communicating branch, draining into the hemiazygos. A small portion of infrarenal IVC blood flowed reversely into the bilateral lumbar veins, via right and left ascending lumbar veins, draining into the azygos vein(right) and hemiazygos vein(left) separately. This bypass explains the expanded LRV, azygos and hemiazygos vein, and bilateral lumbar veins

With the help of free-sectional ultrasound, it was confirmed that her LRV received a large part venous blood from the infrarenal IVC and the right kidney, through an expanded communicating branch, draining into the hemiazygos. A small portion of infrarenal IVC blood flowed reversely into the bilateral lumbar veins vein, via right and left ascending lumbar veins, draining into the azygos vein (right) and hemiazygos vein (left) separately. The moment after her coming to the hospital, the venous blood velocity was 21.5m/s at the left hilum, and 42.2m/s at the right side. In the rest state, the velocity changed to 47.2m/s and 94.2m/s respectively (Additional file 1: Table S2 and Figure S1-3). These changes reflected that the pressure in LRV was higher than the right side and elevated after daily activity. A schematic representation of normal venous system and the variant one is presented in Fig. 2.

The presence of facial and bilateral lower extremity edema was observed upon awakening in the morning, and its severity decreased with physical activity. Additionally, it was noted that both edema and proteinuria worsened during periods of increased exertion, and alleviated during periods of relative inactivity. Based on these observations, we proposed that the IVC interruption resulted in impaired venous reflux in the supine position and contributing to the development of edema in the face and lower extremities.

## Case discussion

To the best of our knowledge, it is the one of the first cases reported of orthostatic proteinuria attributed to subsequent hemodynamic abnormality of IVC interruption in the absence of LRV entrapment and IVC thrombosis. The patient’s LRV received most of infrarenal IVC blood, directly resulting in elevated pressure of LRV and then increasing the pressure of renal venules and efferent arteriole, which caused elevated glomerular capillary hydrostatic pressure and more protein filtering from glomeruli into the primary urine. The pressure of LRV would be more exaggerated due to the calf muscle pump, explaining its postural dependence. Although fixed and reproducible orthostatic proteinuria, this patient had normal serum creatinine and benign renal outcome.

Without longitudinal observation, orthostatic proteinuria is hard to be distinguished from that caused by measuring bias, transient proteinuria after vigorous exercise, and primary or secondary chronic kidney disease (CKD) in this case. After verifying that all quality control (QC) records of urinary microalbumin and total protein were under control, we considered the patient’s highly variant urinary protein was a reflection of her original disease rather than the detection error. With the value of ACR elevated over 400 times after mild to moderate exercise intensity, the degree of fluctuation is much greater than that of postexercise transient proteinuria in healthy subjects [7, 8] and could hardly be explained by chronic kidney disease (CKD). Although the patient had several risk factors, including obesity, hypertension, and multiple cystic in bilateral kidney that could cause secondary kidney diseases [9], the patterns of normal serum creatinine and 24h UTP, plus clear renal structure are not consistent with the characteristics of CKD. Besides, the tests for secondary kidney diseases, comprising of autoimmune antibodies and monoclonal protein were all negative. Therefore, we excluded the possibility of secondary kidney diseases.

Orthostatic proteinuria caused by NCS usually happens in slim young individuals and would alleviate after growing up or gaining weight [10]. The patient was obese and relatively older than those suffered from NCS [1]. As shown in cross sectional CT and ultrasound, her abdominal fat pad prevented the left renal vein from being compressed by the arteries. These features excluded the possibility of NCS. The patient did not exhibit uncommon etiologies of NCS, such as pancreatic neoplasms, retroperitoneal tumor, or lordosis etc. [5]. Rarely, Devarajan reported a 40-year-old female whose orthostatic proteinuria was owing to a kink in LRV [11], the proteinuria was resolved after donating the left kidney to her kid. Two cases of orthostatic proteinuria secondary to suprarenal IVC thrombosis were reported [12]. Similar to the impact of a kink in LRV or the IVC thrombosis, the pressure in LRV and glomerular capillary was elevated in our patient due to IVC interruption.

The development of IVC, happening between the sixth and eighth gestational weeks, is complicated and could be influenced by the various factors. To date various anatomical variations of IVC have been reported. Left IVC with retroaortic right renal vein (RRV), double IVC with retroaortic RRV and circumaortic LRV are types of IVC variations that could present nutcracker phenomenon [6, 13]. The prevalence of infra-hepatic interruption of the IVC is about 0.6% [6]. It might be related to a failure in the development of the right subcardinal-hepatic anastomosis, following the atrophy of the suprarenal IVC. In previous reports of IVC interruption, infrarenal IVC would continue as the azygos vein (right IVC) or as the hemiazygos vein (left IVC) [6, 14]. Our case is unique as her right IVC mainly communicated with hemiazygos vein through LRV and she did not present nutcracker phenomenon. This subtype of IVC interruption has not been reported.

Taken together, the atypical age and body weight, and uncommon IVC abnormality as well as nonspecific presentation contributed to the diagnostic odyssey. Lessons we learned from this patient are that for adult patients with postural proteinuria, abnormality of LRV or IVC except NCS should be examined carefully, combining the ultrasound and radiography. Figuring out the causal relationship is important, because the unexplained but constant proteinuria would lead to anxiety and inappropriate treatment.

Diagnosis and management of this unique case were made with the collective help of the department of nephrology, radiology, ultrasound and clinical laboratory. We suggested that she should stop other drugs and simply continue the use of losartan, as the angiotensin system blocker had been proved to be effective in animal models and clinical cases [15]. The follow-up plans included monitoring kidney function, 24-h urinary protein, and ACR/PCR in first morning excretion and excretion after a fixed-mode activity on the same day in regular intervals. Besides, remaining vigilant to the underlying risk of venous thrombosis and kidney impairment, secondary to vessel abnormality is also important.

Generally, the prognosis of postural proteinuria in young individuals is excellent via several long-term follow-ups [2, 16, 17]. Although the proteinuria in this patient could not be as self-limited as NSC due to the constant IVC variation, her kidney function and daily protein excretion have remained normal for over 30 years, which indicating the benign prognosis, thus the invasive procedures were not necessary. By the last follow-up in Oct 2022, she still had positive protein in urine dipstick analysis of samples she excreted at a clinic near her home.

## Conclusion

It is a rare case of orthostatic proteinuria in an old and obese patient attributed to subsequent hemodynamic aberrations initiated by the IVC interruption without nutcracker phenomenon. For adult with highly variant proteinuria, orthostatic proteinuria caused by renal vein compression or renal vein malformation should be taken into consideration. Orthostatic proteinuria could be confirmed via comparing first morning excretion and spot urine after activity. Elucidation of the etiologies will benefit effective management and prognosis prediction.

## Supplementary Information

**Additional file 1:** **Table S1.** Laboratory results from 2019 to 2021. **Table S2.** Renal venous peak flow velocity. **Figure S1.** The blood flow direction at the junction of LRV and RRV. **Figure S2.** The blood flow nearing the left renal hilum. **Figure S3.** A bundle of blood flow towards one of lumbar veins

## Data Availability

All data related to this study are included in this manuscript and its additional file.

## Abbreviations

24h UTP: 24-Hour urinary total protein
ACR: Albumin-to-creatinine ratio
CKD: Chronic kidney disease
CT: Computed tomography
IVC: Inferior vena cava
LRV: Left renal vein
NCS: Nutcracker syndrome
PCR: Protein-to-creatinine ratio
QC: Quality control
RRV: Right renal vein
