# Supplementary material for: Orthostatic proteinuria due to inferior vena cava interruption without nutcracker phenomenon in an old obese female: a case report and literature review
Source: BMC Nephrol. 2023 Jul 31;24:225. doi: 10.1186/s12882-023-03279-y (PMC10391862; doi:10.1186/s12882-023-03279-y)
Supplement: Supplementary file 1 — Additional file 1: Table S1. Laboratory results from 2019 to 2021. Table S2. Renal venous peak flow velocity. Figure S1. The blood flow direction at the junction of LRV and RRV. Figure S2. The blood flow nearing the left renal hilum. Figure S3. A bundle of blood flow towards one of lumbar veins [file 12882_2023_3279_MOESM1_ESM.docx]

**Table S1. Laboratory results from 2019 to 2021.**

| **Date** | **ACR (mg/g)** | **PCR (mg/g)** | **24hUTP (g)** | **U-PRO**  **(g/L)** | **U-RBC (Cells/μL)** | **Serum Cr (μmoI/L)** |
| --- | --- | --- | --- | --- | --- | --- |
| Jun 19, 2019 | 4131 | - | 0.1 | ≥3.0 | TRACE | - |
| Jul 4, 2019 | 5755 | - | 0.23 | 0.3 | TRACE | - |
| Jul 19, 2019 | 1612 | - | 0.05 | 1 | TRACE | - |
| Aug 2, 2019 | 15 | - | 0.05 | ≥3.0 | TRACE | - |
| Aug 29, 2019 | 115 | - | 0.23 | NEG | TRACE | 56 |
|  | - | - | - | ≥3.0 | TRACE | - |
|  | - | - | - | NEG | NEG | - |
| Dec 10, 2019 | 1219 | 1564 | 0.05 | ≥3.0 | 25 | 59 |
| May 8, 2020 | - | - | 0.13 | 0.3 | NEG | - |
| Jun 18, 2020 | - | - | - | NEG | TRACE | 65 |
| Aug 31, 2020 | - | - | - | ≥3.0 | NEG | 54 |
|  | - | - | 0.05 | 1 | 25 | - |
|  | - | - | - | ≥3.0 | TRACE | - |
| Mar 15, 2021 | 1794 | 2422 | 0.1 | 1 | NEG | 62 |

ACR, albumin-creatinine ratio; PCR, protein-creatinine ratio; 24hUTP, 24-hour urinary total protein; U-PRO, U-RBC, urinary protein and red blood cell detected by dry chemistry method; Cr, creatinine.

**Table S2. Renal venous peak flow velocity**

| **Detection Part** | **Velocity of RRV (m/s)** | **Velocity of LRV (m/s)** |
| --- | --- | --- |
| Resting state |  |  |
| IVC | 63.8 | 35.7 |
| Near renal hilum | 73.6 | N.T. |
| Renal hilum | 94.2 | 47.2 |
| After activity |  |  |
| IVC | 26 | 24 |
| Renal hilum | 42.2 | 21.5 |

In the resting state, the velocity of LRV was 35.7m/s and 47.2m/s at the proximal and distal left renal hilum, lower than that of right side which was 63.8m/s and 94.2m/s. The moment right after her coming to the hospital, the velocity changed to 24m/s and 21.5m/s at proximal and distal part of the left hilum, and the number was 26m/s and 42.2m/s at the right side. The velocity of left hilum after exercise is the lowest, representing its highest resistance. IVC, inferior vena cava. N.T., not test.

| 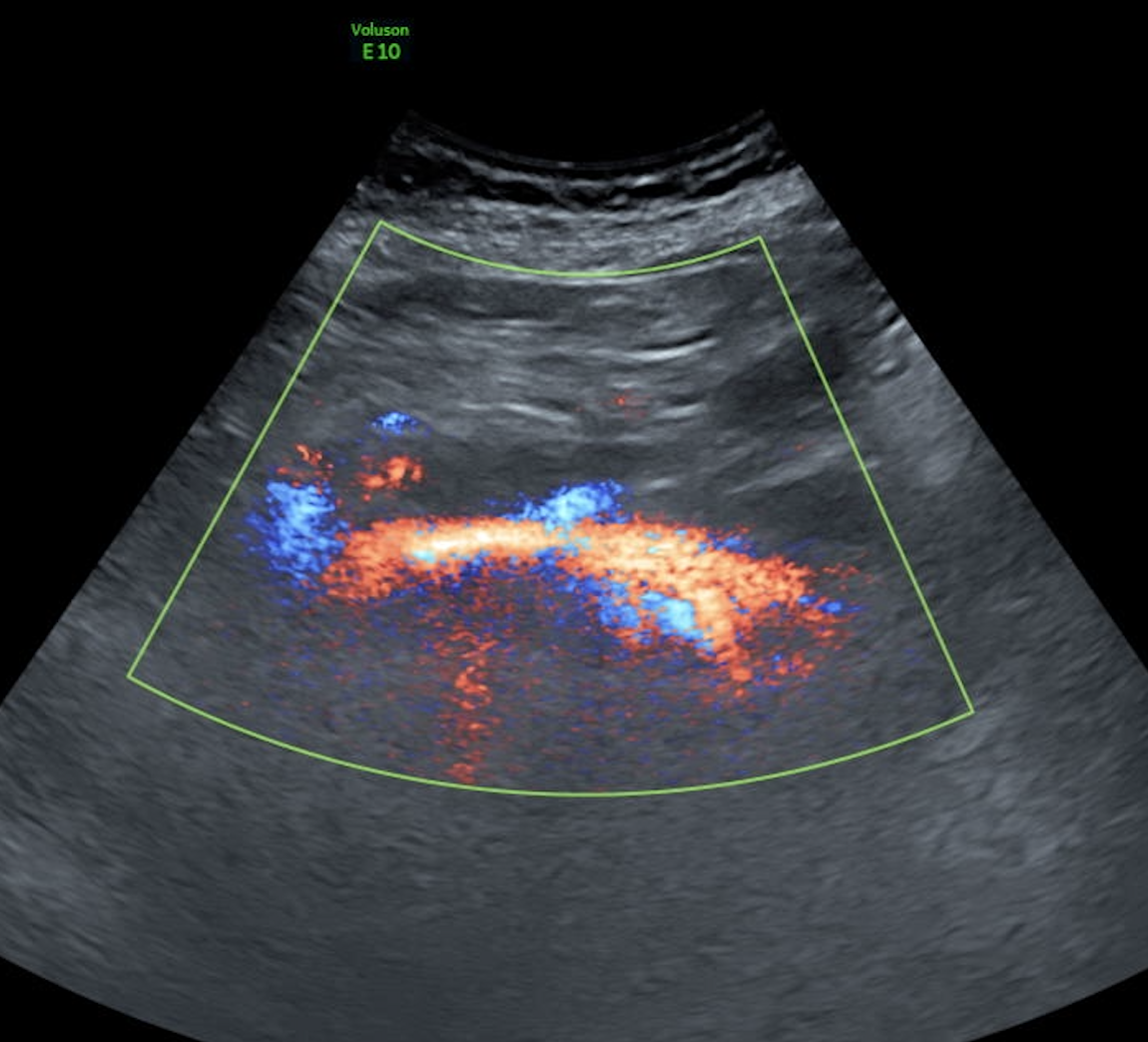 |
| --- |
| Figure S1. The blood flow direction at the junction of LRV and RRV. At the junction of LRV and RRV, the blood flow direction was from right to left, as shown by power Doppler. LRV, left renal vein; RRV, right renal vein. |

| 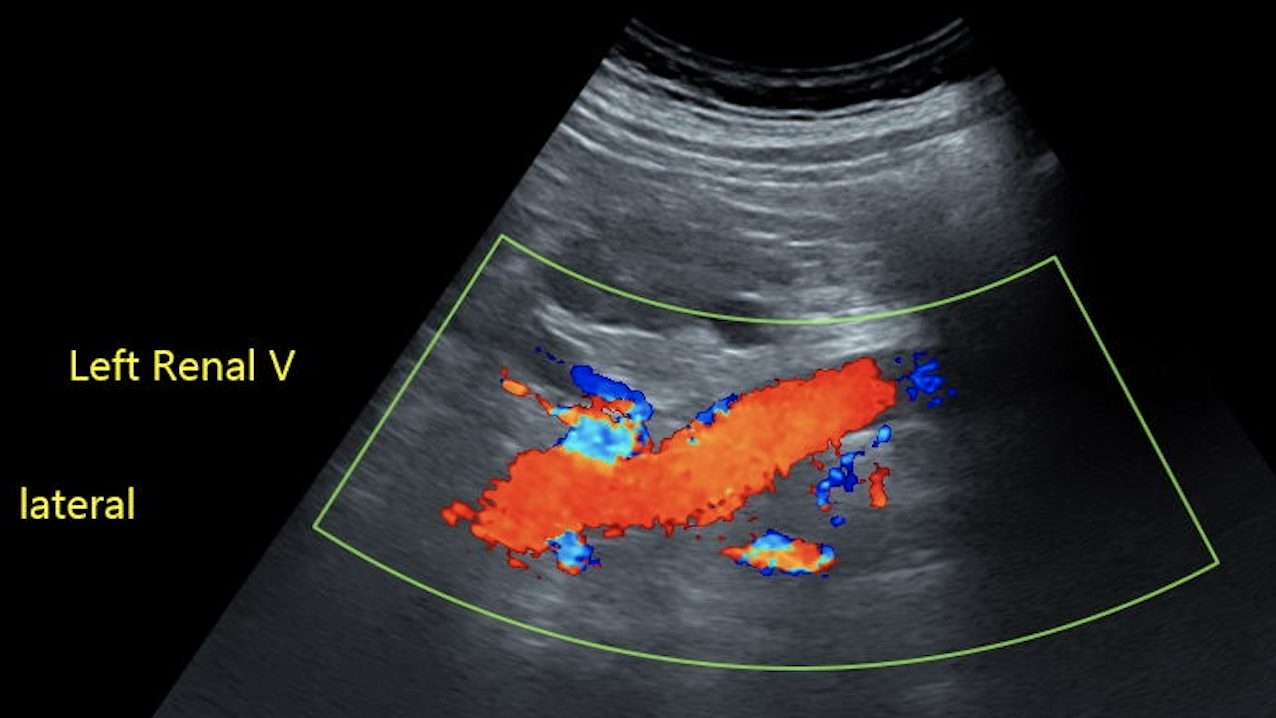 |
| --- |
| Figure S2. The blood flow nearing the left renal hilum. Nearing the left renal hilum, a bundle of blood flow originated from the LRV drained into a communicating vessel consistent with the vessel marked by % in Figure 1. LRV, left renal vein. |

| 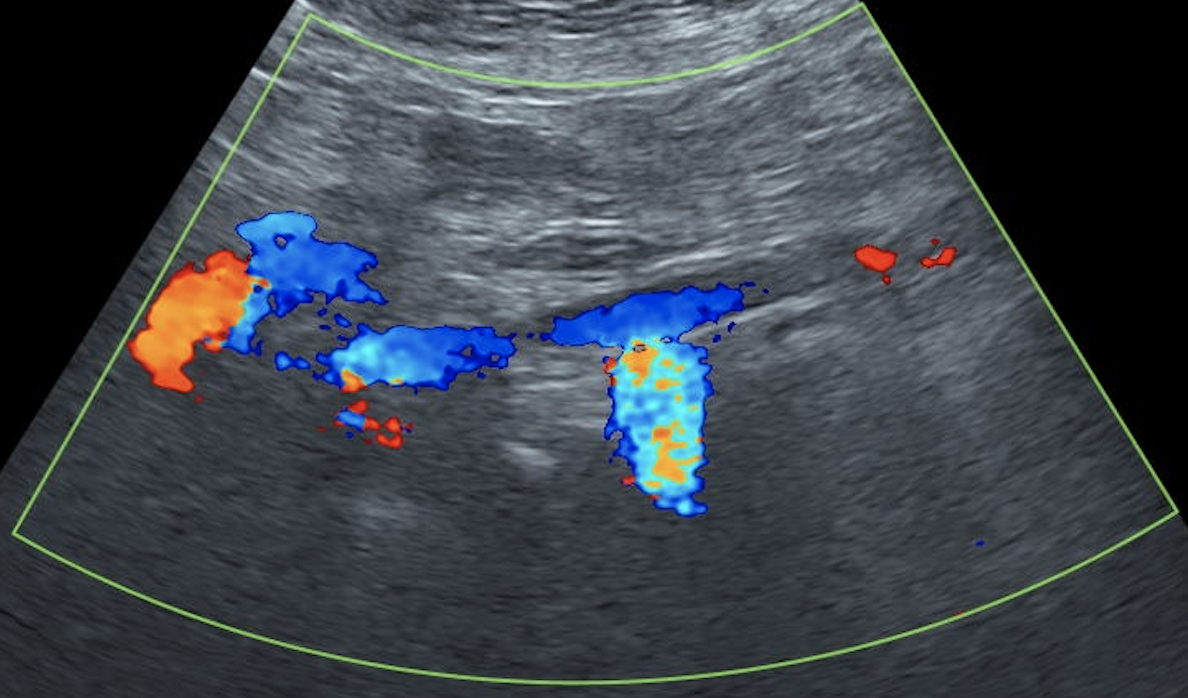 |
| --- |
| Figure S3. A bundle of blood flow towards one of lumbar veins. At one part of the IVC below the level of the RRV, there is a bundle of gross blood flow signal which indicated that the IVC drained into the lumbar veins reversely. IVC, inferior vena cava. RRV, right renal vein. |
